# Interleukin-1 regulates myeloid cell trafficking and cerebral blood flow following intracerebral haemorrhage

**DOI:** 10.1242/dmm.052306

**Published:** 2025-09-30

**Authors:** Jack Barrington, Jack Rivers-Auty, Patrick Strangward, Sabrina Tamburrano, Nikolett Lénárt, Tessa Swanton, Eloise Lemarchand, Adrian R. Parry-Jones, Ádám Dénes, David Brough, Stuart M. Allan

**Affiliations:** ^1^ Division of Neuroscience, School of Biological Sciences, Faculty of Biology, Medicine and Health, The University of Manchester, Manchester Academic Health Science Centre, AV Hill Building, Manchester M13 9PT, UK; ^2^ Geoffrey Jefferson Brain Research Centre, Manchester Academic Health Science Centre, Northern Care Alliance NHS Foundation Trust, The University of Manchester, Manchester M13 9PT, UK; ^3^ School of Medicine, College of Health and Medicine, University of Tasmania, Hobart, Tasmania 7001, Australia; ^4^ Lydia Becker Institute of Immunology and Inflammation, Faculty of Biology, Medicine and Health, Manchester Academic Health Science Centre, The University of Manchester, Manchester M13 9PT, UK; ^5^ “Momentum” Laboratory of Neuroimmunology, HUN-REN Institute of Experimental Medicine, Szigony u. 43, Budapest 1083, Hungary; ^6^ Division of Cardiovascular Sciences, Faculty of Biology, Medicine and Health, The University of Manchester, Manchester Academic Health Science Centre, Manchester M13 9PT, UK

**Keywords:** Intracerebral haemorrhage, Stroke, Interleukin-1, Brain inflammation, Neuroinflammation

## Abstract

Intracerebral haemorrhage (ICH) is a devastating stroke subtype lacking effective therapies. Understanding key pathological processes related to acute brain damage will help deliver better outcomes for ICH. Herein, we provide evidence that myeloid cell trafficking to the parenchyma is a conserved feature of ICH in clinical and experimental settings. Consistent with others, we show that monocytes contribute to acute brain damage following collagenase-induced murine ICH. Using RNA sequencing, we identified the pro-inflammatory cytokine interleukin-1 (IL-1) as a potential upstream regulator of the acute inflammatory response, with histological data pinpointing mononuclear phagocytes as the principal cellular source of IL-1 in patient and animal tissue. In agreement, inhibition of IL-1 receptor 1 (IL-1R1) with IL-1 receptor antagonist reduced recruitment of myeloid cells. However, IL-1R1 inhibition also worsened neuromotor outcomes and reduced cerebral blood flow to the affected hemisphere. Thus, we reveal dichotomous actions of IL-1-dependent inflammation following brain haemorrhage. Although IL-1 regulates myeloid cell trafficking, it also appears to regulate cerebral blood flow. Therefore, further investigation into the consequences of IL-1 signalling following brain haemorrhage is required to clarify future therapeutic options.

## Teaser

**Summary:** Interleukin-1 has complex positive and negative functions during acute intracerebral haemorrhage; although it regulates myeloid cell trafficking, it also appears to regulate cerebral blood flow.

## INTRODUCTION

Stroke is one of the leading causes of death and disability worldwide. There are two main subtypes of stroke. Ischaemic stroke (IS) caused by occlusion of a cerebral artery accounts for ∼65% of all stroke incidents worldwide (Feigin et al., 2025). Intracerebral haemorrhage (ICH) accounts for ∼29% of worldwide stroke incidents and results from a cerebral vessel rupturing and blood entering brain parenchyma. Despite causing ∼50% fewer strokes, ICH leads to a roughly equivalent amount of deaths and disability-adjusted life years lost as IS (Feigin et al., 2025). A lack of effective therapies for ICH patients is one notable force driving these disproportionate impacts. Therefore, understanding the biological pathways of brain damage following ICH will help develop future treatments to improve ICH outcome.

Research in ICH indicates two temporally distinct waves of injury occur (Qureshi et al., 2009). Mass effect, driven by intraparenchymal accumulation of blood products, causes initial neuronal injury, but is followed by a progressive secondary injury accompanied by the onset of inflammation. Tissue injury releases damage associated molecular patterns (DAMPs), triggering inflammation through toll-like receptor (TLR) activation (Liesz et al., 2011; Sansing et al., 2011b). TLR signalling (e.g. TLR4) leads to production of pro-inflammatory cytokines that amplify local inflammation and recruit circulating immune cells. Microglia, a brain-resident population of immune cells, show a diverse range of responses to haemorrhage that may either contribute to ongoing injury or mediate repair (Lan et al., 2017; Mastorakos et al., 2021). Circulating immune cells contribute to acute brain damage through release of matrix metalloproteinases, reactive oxygen species and cytokines. Preventing either monocytes (Hammond et al., 2014a) or neutrophils (Sansing et al., 2011a) reaching the brain improves outcome in animal models of ICH. Increased levels of monocyte and neutrophil activity are also linked to poor outcome after ICH in humans (Hammond et al., 2014a; Shi et al., 2022). However, there are no pharmacological strategies targeting acute recruitment of neutrophils and monocytes during ICH.

Vascular inflammation is important in myeloid cell trafficking during ICH (Hammond et al., 2014b). The prototypical pro-inflammatory cytokine interleukin (IL)-1, a downstream product of TLR4, triggers myeloid cell recruitment to the brain through actions on the cerebral vasculature (Liu et al., 2019). IL-1 promotes myeloid cell infiltration (Wong et al., 2019), exacerbates excitotoxic damage (Lawrence et al., 1998) and contributes to the no-reflow phenomenon (Murray et al., 2014) during IS; however, its influence in ICH remains an important open question given high quality evidence of pathway activation in patient brain tissue (Loan et al., 2022).

Here, we provide evidence that mononuclear phagocytes (MNPs) produce IL-1 following ICH and signalling through the type 1 IL-1 receptor (IL-1R1) is required for central monocyte and neutrophil recruitment. Despite this, and in contrast to findings in other studies, IL-1 inhibition worsens neuromotor dysfunction and this is associated with decreased cerebral blood flow (CBF), through as yet undefined mechanisms. We thus uncover opposing functions of IL-1-dependent neuroinflammation in the first 24 h of ICH.

## RESULTS

### Genes related to innate inflammation are enriched in the brain following haemorrhage

**Fig. 1.**
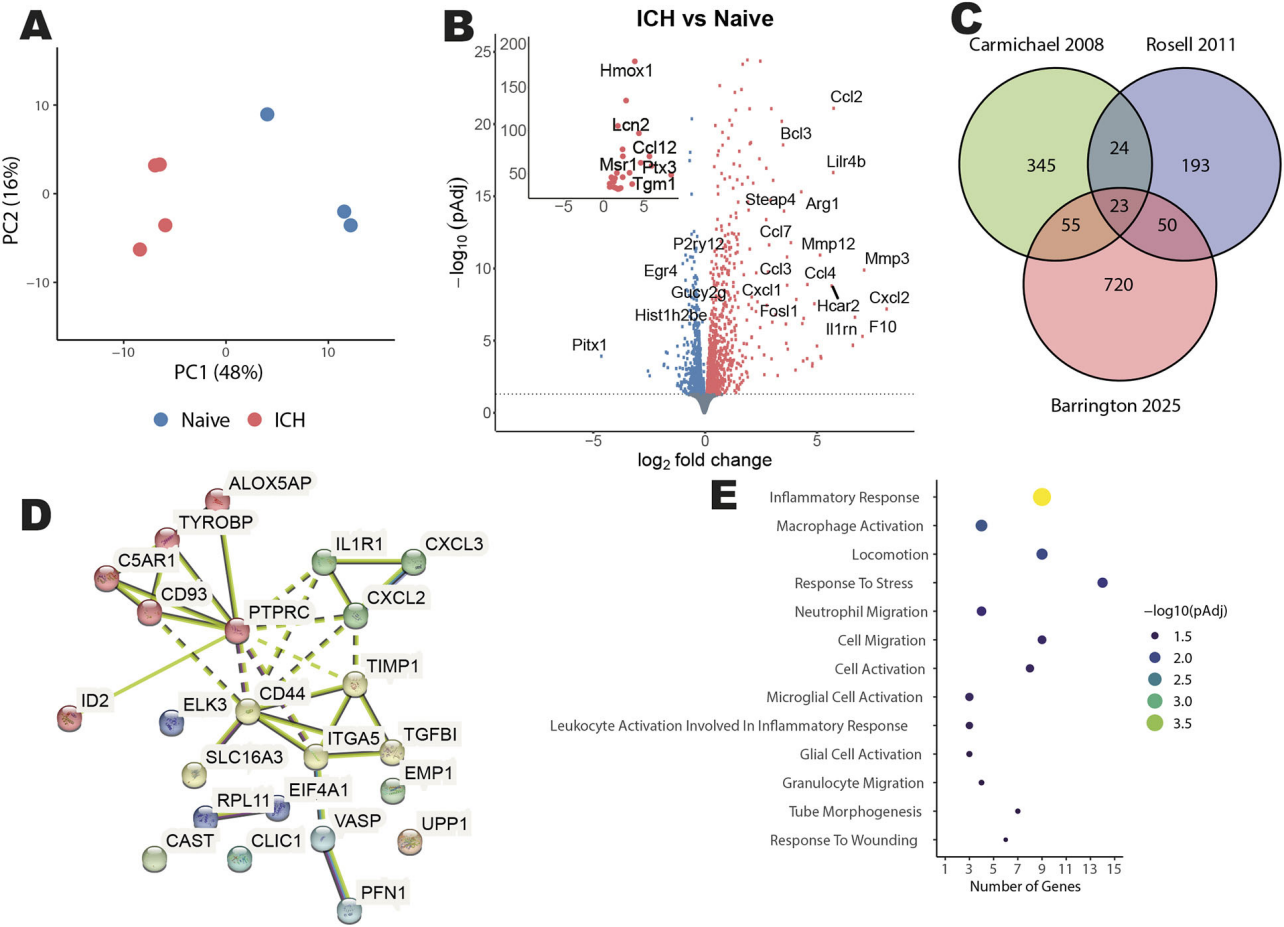
**Intracerebral haemorrhage-regulated gene changes implicate leukocyte recruitment as the dominant feature of acute phases.** RNA sequencing was performed on RNA extracted from a single brain hemisphere of naïve mice (*n*=3) and ipsilateral hemispheres of mice subjected to intracerebral haemorrhage (ICH) 24 h previously (*n*=4). (A) Unguided principal component analysis of transcriptional data. Blue, naïve animals; red, ICH animals. (B) Volcano plot showing genes significantly enriched in naïve animals (blue) and those significantly enriched following brain haemorrhage (red). PAdj, adjusted *P*-value. (C) Venn diagram visualising genes enriched across two previous studies of human perihaematomal tissue (Carmichael et al., 2008; Rosell et al., 2011) and our mouse data (‘Barrington 2025’). (D) Protein–protein interaction network of the 23 enriched molecules found across all studies in C, clustered using the Markov Clustering Algorithm; cluster family is represented by node colour, dashed line edges define cluster boundaries. Edge colour represents interaction data source: cyan and magenta, known interactions; red, green and blue, predicted interactions; lime, black and light blue, other interactions. (E) Bubble plot containing functional enrichment results of the 23 molecules compared across Gene Ontology Biological Processes terms.

To understand the cellular and molecular changes following acute ICH, we performed RNA sequencing (RNA-seq) on RNA extracted from the right (ipsilateral) hemisphere of control mice or mice injected with collagenase to model ICH 24 h prior to cull (Kirkman et al., 2011). Unguided principal component and differential expression analysis revealed stark differences in the transcriptional landscape of ICH and control animals (Fig. 1A,B). Gene set enrichment analysis (GSEA) revealed that transcriptional profiles in ICH were related to inflammatory biological processes (e.g. cytokine production, myeloid cell chemotaxis and macrophage activation), whereas those in control brains were related to central nervous system physiology (e.g. synapse biology, axon guidance and neurotransmitter activity) (Fig. S1A). Accordingly, of the 1278 differentially expressed genes, a large proportion of those upregulated following ICH were attributed to inflammation (such as *Ccl2*, *Icam1* and *Il1b*), whereas those downregulated were linked to neuronal pathways (such as *Bdnf*, *Grin2b* and *Homer2*) (Fig. S1B). We compared the genes enriched in ICH samples in our data with those enriched in perihaematomal tissue from human cases in two previous publications (Carmichael et al., 2008; Rosell et al., 2011) (Fig. 1C). Twenty-three genes were enriched in our data and both of the previous datasets (Carmichael et al., 2008; Rosell et al., 2011). These 23 genes contained markers of microglial/macrophage activation (such as *Tyrobp*, *Ptprc* and *C5ar1*), neutrophil-trafficking molecules (such as *Il1r1*, *Cxcl3* and *Cxcl2*), alongside markers of cell migration (such as *Vasp*, *Pfn1* and *Itga5*). Protein–protein interaction network clustering identified similar molecular clustering patterns (Fig. 1D). Functional enrichment on biological processes indicated that the 23 genes enriched across all studies were indeed enriched in pathways such as inflammation, macrophage activation, granulocyte migration and response to tissue stress (Fig. 1E). Collectively, these data show that diverse inflammatory pathways are engaged in the brain following ICH.

### Peripheral immune cells accumulate in the brain following ICH

**Fig. 2.**
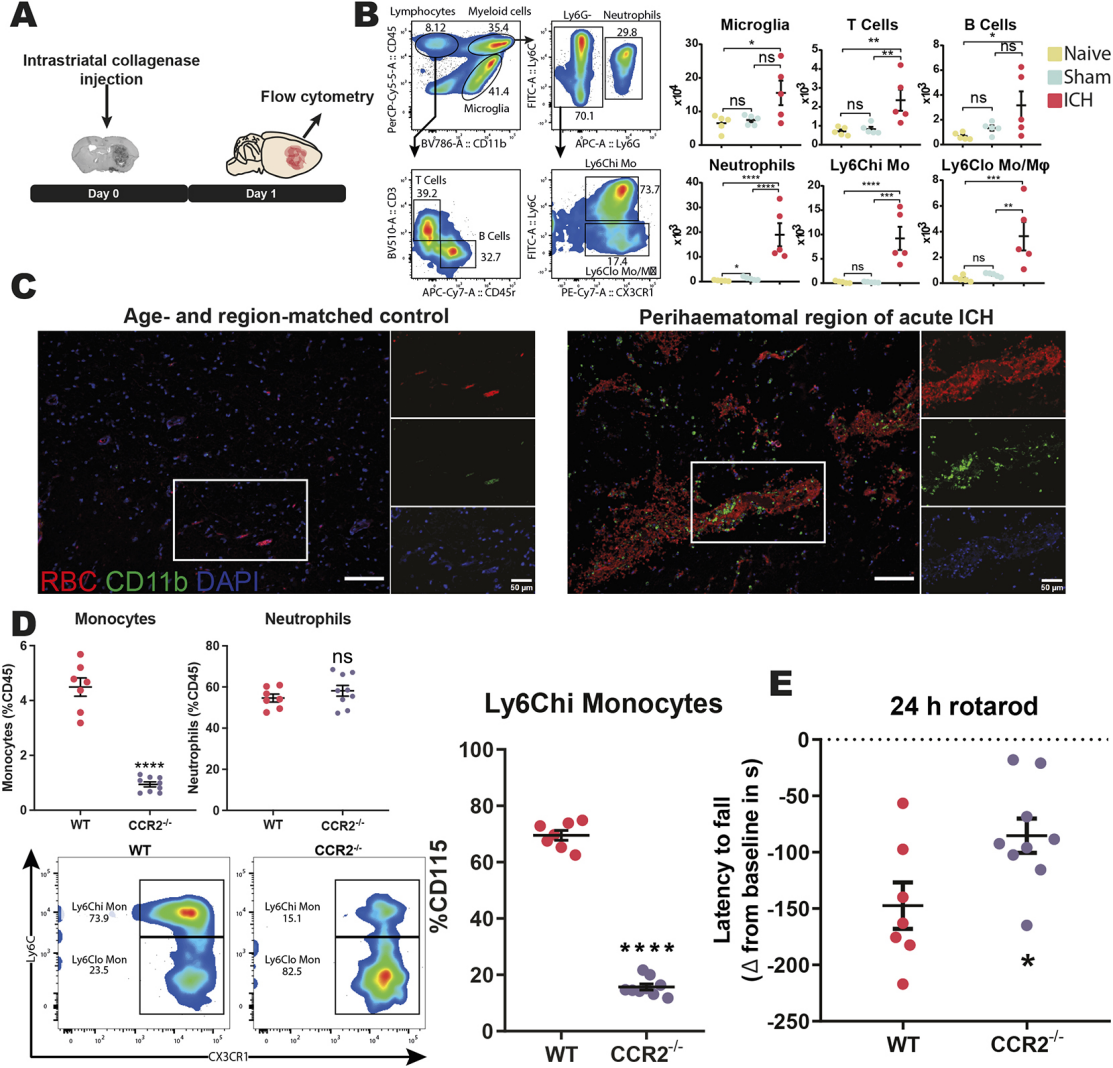
**Myeloid cells dominate the acute response to ICH and contribute to early damage.** (A) Single cells were isolated from the right hemisphere of naïve, sham-operated and collagenase-induced ICH mice 24 h post-surgery and immunophenotyped using flow cytometry. (B) Left: gating strategy used during flow cytometry analysis. Right: cell counts are shown from the following populations: CD45^int^ CD11b^+^ microglia, CD45^hi^ CD11b^+^ Ly6G^+^ neutrophils, CD45^hi^ CD11b^+^ Ly6G^−^ Ly6C^hi^ monocytes (Mo), CD45^hi^ CD11b^+^ Ly6G^−^ Ly6C^lo^ monocytes/macrophages (Mϕ), CD45^hi^ CD11b^−^ CD45R^+^ B cells, and CD45^hi^ CD11b^−^ CD3^+^ T cells. Data presented as mean+s.e.m., *n*=5, two independent experiments. ns, not significant; *P<0.05; **P<0.01; ***P<0.001, ****P<0.0001, determined by one-way ANOVA with Tukey's post-hoc test. (C) Formalin-fixed paraffin-embedded post-mortem human brain tissue from age- and region-matched control (left) and ICH (right) cases were immunostained for the myeloid cell marker CD11b (green) and DAPI (blue), with red blood cell (RBC) autofluorescence shown in red. One representative image from three cases per group shown. Scale bars: 100 µm (50 μm in insets). (D) White blood cells were isolated from whole blood of wild-type (WT) (*n*=7) and chemokine receptor 2 (*Ccr2*)^−/−^ (*n*=9) male littermate mice. Top left: CD115^+^ monocytes and Ly6G^+^ neutrophils as a percentage of all CD45^+^ immune cells. Bottom left: representative flow plots of the circulating monocyte compartment of each genotype. Right: the amount of Ly6C^hi^ monocytes as a percentage of total circulating monocytes from each genotype. ns, not significant; ****P<0.0001, determined by unpaired two-tailed *t*-test. (E) Following a 3 day training period on the rotarod assay, WT and *Ccr2*^−/−^ littermates were subjected to collagenase-induced ICH and tested on the rotarod again 24 h later. Data presented as net difference in latency to fall from baseline performance, *n*=7-9, four independent experiments. *P<0.05, determined by unpaired two-tailed *t*-test.

Having identified changes to the molecular inflammatory landscape following ICH, we subsequently performed flow cytometry to assess changes to immune cell composition. Microglia are the only immune cell inhabiting the brain parenchyma during steady state. However, activation of cerebral vasculature and breakdown of the blood–brain barrier facilitates ingress of peripheral immune cells during disease (Anrather and Iadecola, 2016). In line with this, compared to naïve and sham-operated controls, animals with ICH had more T cells, neutrophils, Ly6C-high (Ly6C^hi^) and Ly6C-low (Ly6C^lo^) monocytes/macrophages recruited to the ipsilateral hemisphere 1 day post-surgery (Fig. 2A,B). Myeloid cells, specifically neutrophils and Ly6C^hi^ monocytes, increased by the greatest magnitude (∼20-fold) compared to those in sham-operated animals.

Similarly, we observed accumulation of CD11b^+^ (also known as ITGAM^+^) myeloid cells specifically in acute ICH human brain samples using immunohistochemistry (Fig. 2C). Age- and region-matched control brain tissue was notably absent of extravasated red blood cells (RBCs) and immunostaining for CD11b. However, CD11b^+^ cells were observed within and around vessels with perivascular RBC accumulation in ICH brain tissue from cases that died within the first 3 days of onset. CD11b^+^ cells were also observed within the parenchyma in ICH tissue and in regions of haemorrhage pathology of a cerebral small vessel disease (cSVD) tissue cohort (Fig. S2).

These data show that myeloid cell trafficking to the brain occurs in both human and mouse ICH, with broader profiling tools in mouse revealing that neutrophils and Ly6C^hi^ monocytes predominate.

### Monocytes contribute to acute neuromotor deficits during haemorrhage

The recruitment of monocytes to inflamed brain tissue contributes to acute injury in various disease settings, including ICH (Hammond et al., 2014a). The efflux of monocytes from the bone marrow relies on signalling through the monocyte-expressed receptor CCR2 (Boring et al., 1997). Here, we used CCR2-deficient mice to assess the impact of ablating monocyte trafficking to brains following ICH. Flow cytometry profiling of circulating immune cells established that *Ccr2*^−/−^ animals had ∼4-fold reduction in monocytes compared to *Ccr2^+/+^* controls, but no difference in the amount of circulating neutrophils (Fig. 2D). Ly6C^hi^ monocytes were specifically reduced in number in *Ccr2*^−/−^ mice. One day post-ICH, *Ccr2*^−/−^ mice performed significantly better on the rotarod assay than wild-type littermates (Fig. 2E). Thus, preventing monocyte efflux from the bone marrow by genetic deletion of *Ccr2* limits neuromotor deficits following ICH; however, it is not clear which molecule(s) govern their entry to brain parenchyma and whether these can be targeted therapeutically.

### IL-1 is important for myeloid cell trafficking to the haemorrhaged brain

Having established myeloid cell recruitment as a potential therapeutic target in acute ICH, we next looked to identify the cellular/molecular processes governing this. GSEA on published cell-specific gene sets revealed that gene signatures elevated in ICH samples from our RNA-seq data (Fig. 1) strongly correlated with a number of published macrophage signatures, including Kupffer cells, microglia and other brain macrophages (Fig. S3A,B). Macrophage activation was also one of most significantly enriched biological processes (Fig. S3A-C). We thus reasoned that microglia, the brain-resident macrophages, first respond to ICH and release factors to recruit circulating myeloid cells (focusing here predominantly on neutrophils). However, despite efficiently depleting microglia with the CSF1R antagonist PLX5622, we found no effect of this treatment on neutrophil recruitment to the brain 1 day after ICH (Fig. S3D). To avoid functional redundancy between cell classes, we subsequently focused our efforts on identifying key molecular processes promoting myeloid cell recruitment.

**Fig. 3.**
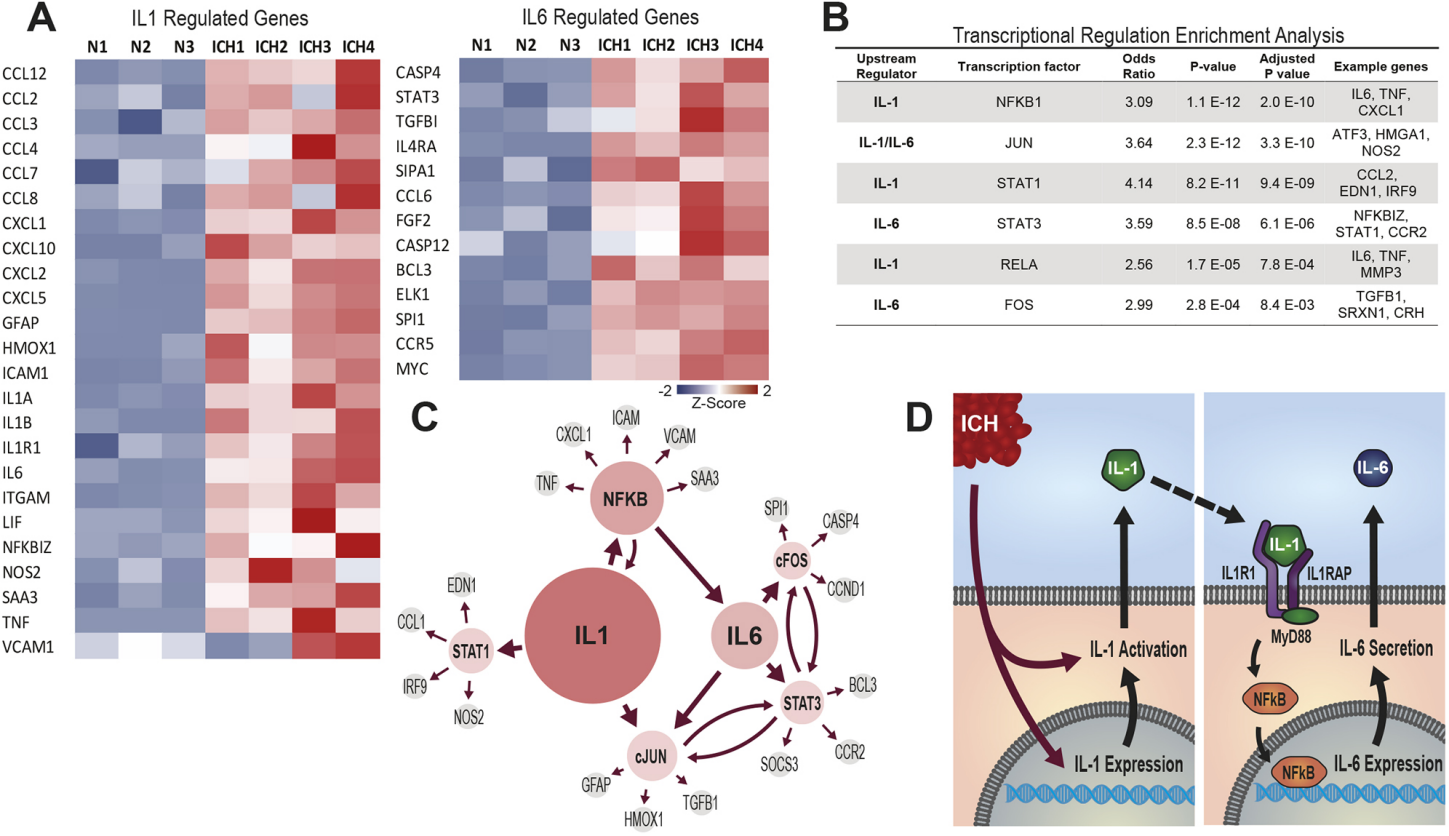
**Transcriptional profiling implicates the IL-1–IL-6 axis as central feature of neuroinflammation following ICH.** Data from Fig. 1 were further analysed to evaluate potential regulators of myeloid cell recruitment. (A) Heatmaps of the top significant genes identified within the most notable networks: IL-1 (left) and IL-6 (right). (B) Transcriptional regulation enrichment analysis. (C) Cluster and pathway analyses identified IL-1β and IL-6 signalling as the predominant pathways regulating inflammation following ICH. Areas of the coloured circles are proportional to the number of significantly upregulated genes identified downstream of the labelled regulator molecule. IL-1 was the dominant signalling pathway identified, with 209 downstream genes found to be upregulated. (D) Proposed signalling cascade responsible for the substantial inflammatory response seen following ICH.

IL-1 can mediate neuroinflammation, and many of the differentially expressed genes following ICH were transcription factor targets for sensors of IL-1 and IL-6 (*Nfkb1*, *Jun*, *Stat1*, *Stat3*, *Rela* and *Fos*) (Fig. 3A,B). In fact, most of the upregulated inflammatory genes were part of the IL-1 and IL-6 pathways, and the IL-1 receptor was enriched across our mouse data and both of the compared human datasets, where it sat in a protein–protein interaction hub with key myeloid cell trafficking chemokines (Fig. 1D and Fig. 3A). Network analysis further suggested both IL-1 and IL-6 as possible upstream regulators in the inflammatory response to ICH (Fig. 3C). Of the two molecules, IL-1 was associated with the greatest amount (209) of differentially expressed genes, and both clinical and preclinical evidence indicates that IL-6 sits downstream of IL-1R1 activation (Lévesque et al., 2016; Smith et al., 2018) (Fig. 3D). These data, therefore, suggested that IL-1 is a potential upstream regulator of brain inflammation following ICH.

**Fig. 4.**
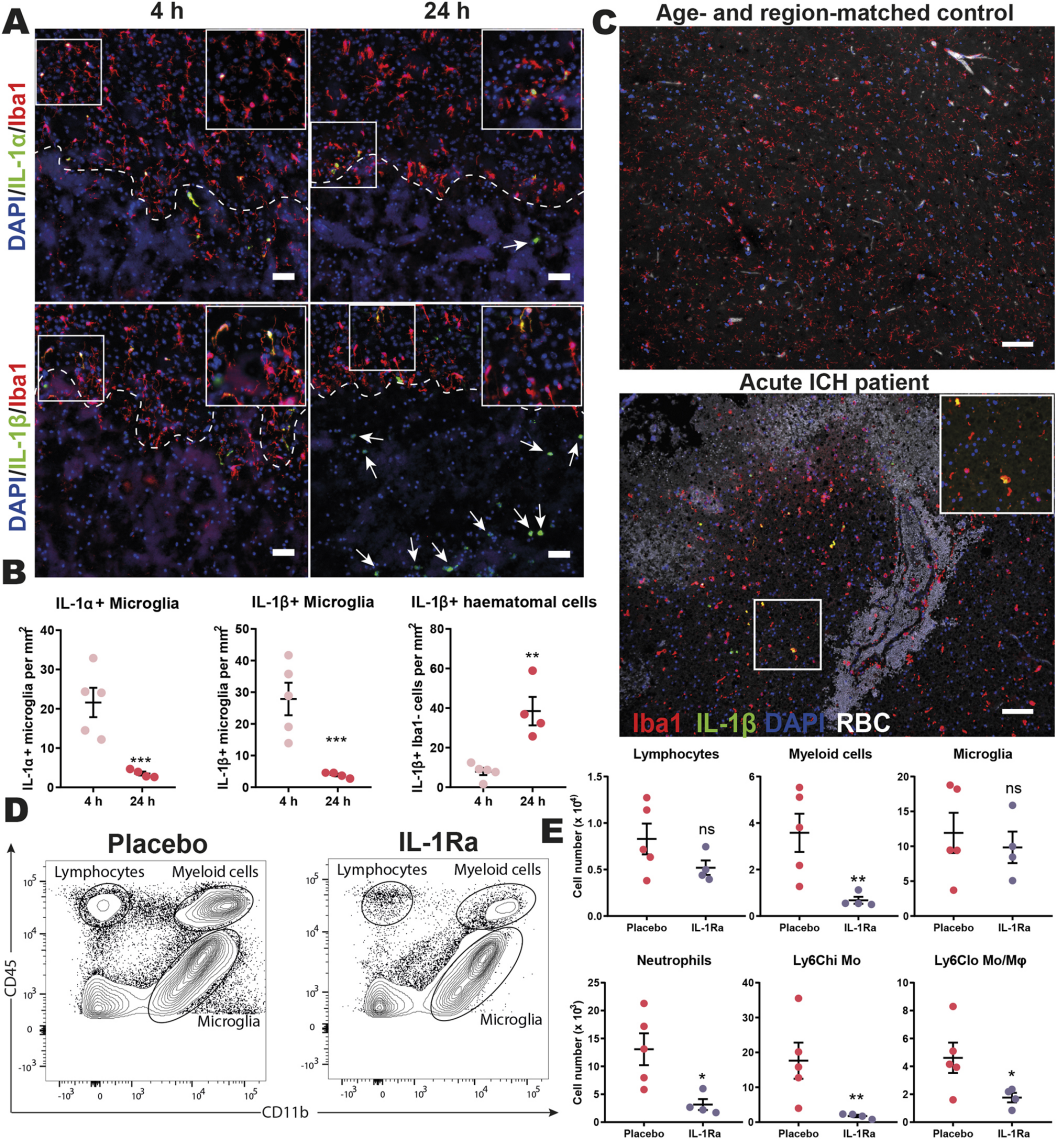
**IL-1 produced by mononuclear phagocytes controls acute recruitment of myeloid cells during ICH.** (A) Mice were subjected to collagenase-induced ICH and culled at 4 h (*n*=5) and 24 h (*n*=4) post-ICH. 20 µm coronal brain sections were immunostained for either IL-1α (green, top row) or IL-1β (green, bottom row) together with Iba1 (red) and DAPI (blue). Dashed lines represent haematoma border, arrows define haematomal IL-1β+ cells, and insets are higher-magnification images of respective white boxes. Scale bars: 50 µm. (B) Quantification of IL-1^+^ cells. (C) 8 µm sections of formalin-fixed paraffin-embedded post-mortem human brain tissue from age- and region-matched control (top) and acute ICH (bottom) cases were immunostained for Iba1 (red), IL-1β (green) and DAPI (blue). Red blood cell (RBC) autofluorescence can be seen in white. One representative image from three patients per group is shown. Scale bars: 100 µm. Insets are higher-magnification images of the areas within the white boxes. (D) Representative flow plot of cells isolated from brains of mice injected with central (10 µg intrastriatal) and peripheral (100 mg kg^−1^ subcutaneous) IL-1 receptor antagonist (IL-1Ra) (*n*=4) or placebo (*n*=5) and subjected to ICH, two independent experiments. (E) Cell counts are shown from the following populations: CD45^hi^ CD11b^−^ lymphocytes, CD45^int^ CD11b^+^ microglia, CD45^hi^ CD11b^+^ myeloid cells, CD45^hi^ CD11b^+^ Ly6G^+^ neutrophils, CD45^hi^ CD11b^+^ Ly6G^−^ Ly6C^hi^ monocytes (Mo), CD45^hi^ CD11b^+^ Ly6G^−^ Ly6C^lo^ monocytes/macrophages (Mϕ). Data presented as mean+s.e.m. ns, not significant; *P<0.05, **P<0.01, ***P<0.001, determined by unpaired two-tailed *t*-test.

The IL-1 family has two main related pro-inflammatory agonists, IL-1α and IL-1β, that signal through IL-1R1. In the brain, IL-1 is reported to control monocyte and neutrophil trafficking and directly contribute to damaging inflammation after injury (Liu et al., 2019). However, a specific role for IL-1 in ICH remains undefined. Using immunohistochemistry, we observed both IL-1α and IL-1β production by perihaematomal microglia within 4 h of inducing ICH in mice (Fig. 4A,B). IL-1β was also produced by small round cells present within the haematoma that increased in number over the first day, and flow cytometry analysis indicated that these cells were, in fact, infiltrating MNPs, as only CD45^hi^ CD11b^+^ Ly6G^−^ cells expressed greater amounts of IL-1β than shams (Fig. 4A,B; Fig. S4A). Similarly, IL-1β was observed in Iba1^+^ (also known as AIF1^+^) cells in clinical acute ICH samples and in smaller haemorrhages attributed to cSVD (Fig. 4C; Fig. S4B).

IL-1 receptor antagonist (IL-1Ra) was used to assess the functional effects of IL-1 from the onset of ICH. To block IL-1 from central and peripheral MNP compartments, an intrastriatal bolus of 10 μg IL-1Ra was administered immediately before collagenase injection, followed by an immediate subcutaneous 100 mg kg^−1^ dose, which was repeated 6 h later. Using flow cytometry (Fig. 4D,E) and immunohistochemistry (Fig. S4C) 24 h post-ICH, we observed fewer myeloid cells in the brains of animals injected with IL-1Ra. It is hypothesised that IL-1 facilitates myeloid cell trafficking during neuroinflammation by promoting vascular inflammation (Liu et al., 2019; Wong et al., 2019). In line with this, fewer vascular cells expressed the inflammatory adhesion molecule VCAM1 in the perihaematomal region of IL-1Ra-treated mice (Fig. S4D).

IL-1β is produced as an inactive precursor molecule that is activated upon the formation of macromolecular complexes called inflammasomes, which result in activation of the IL-1β-activating enzyme caspase-1 (Schroder and Tschopp, 2010). Some studies suggest that inflammasome activation has a causal role in myeloid cell recruitment during brain haemorrhage (Barrington et al., 2017; Ma et al., 2014; Ren et al., 2018). However, injection with the specific caspase-1 inhibitor VX-765, under the same treatment regimen as for the IL-1Ra experiments, had no effect on immune cell trafficking in the first 24 h post-ICH (Fig. S4F). VX-765 showed efficacy in blocking inflammasomes *in vitro*, but we lack validation of its actions *in vivo* (Fig. S4E). Collectively, these data reveal that IL-1 is a key regulator of myeloid recruitment following brain haemorrhage.

### IL-1 blockade limits neuromotor recovery and exacerbates haemorrhage-induced reductions in CBF

**Fig. 5.**
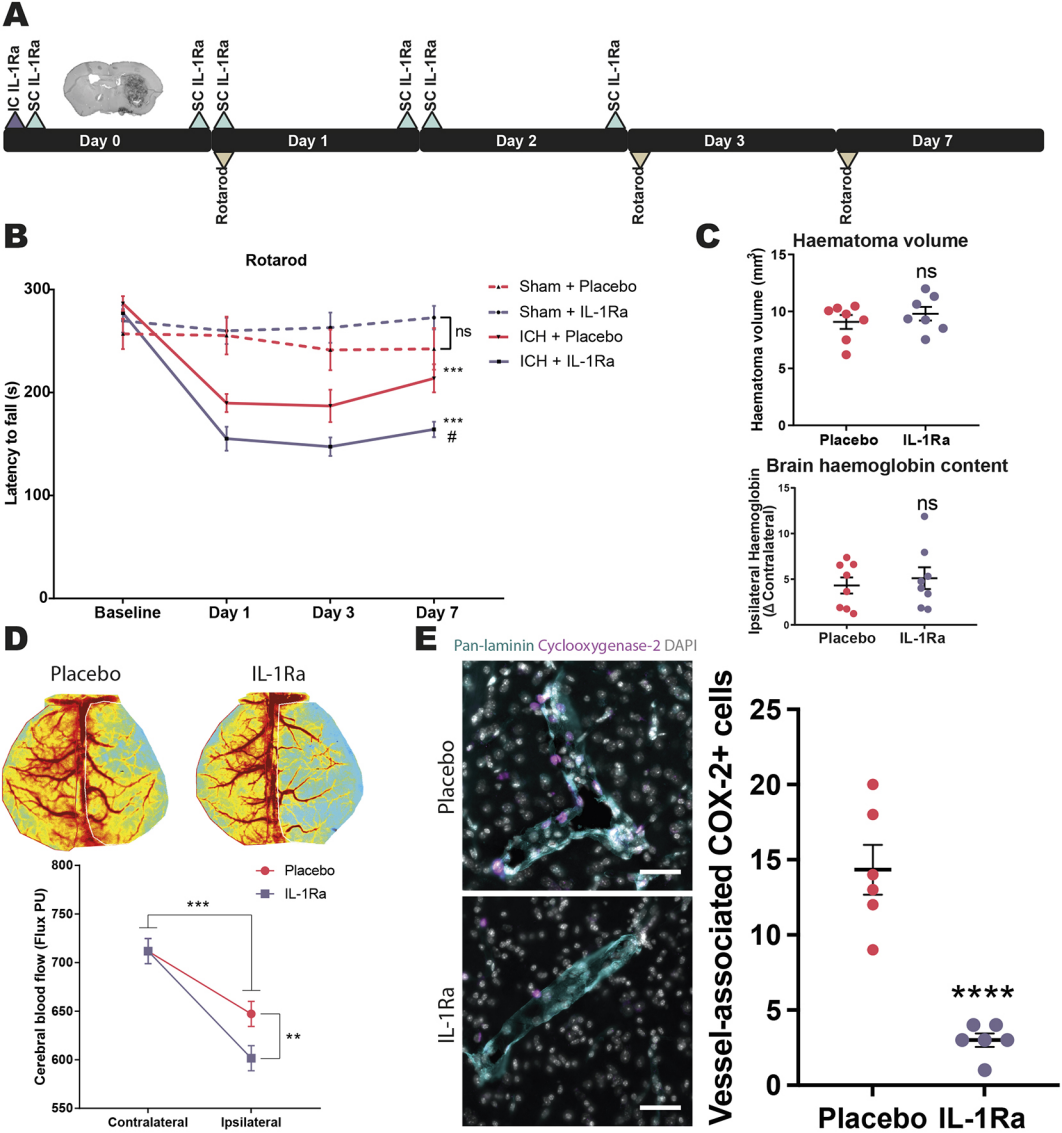
**IL-1 inhibition worsens neuromotor injury and decreases blood flow to sites of ICH.** (A) Experimental design consisted of a placebo or an IL-1 receptor antagonist (IL-1Ra) treatment regimen of 10 µg intrastriatal (IC) injection followed by a 100 mg kg^−1^ subcutaneous (SC) dose, prior to ICH induction, followed by another 100 mg kg^−1^ subcutaneous dose 6, 24, 30, 48 and 54 h later. Rotarod performance was also measured at day 1, 3 and 7. (B) Rotarod performance of placebo (*n*=7) or IL-1Ra (*n*=6)-treated sham and ICH mice. ns, not significant; ***P<0.0001 compared to relative sham, ^#^*P*<0.05 compared to ICH+placebo, determined by mixed-effects model. Four independent experiments. (C) Haematoma volume (*n*=7) and haemoglobin content (*n*=8) was assessed using histological (top) and molecular (bottom) analysis of brains. Data presented as mean±s.e.m. ns, not significant, determined by unpaired two-tailed *t*-test. (D) Cerebral blood flow was quantified using laser speckle contrast imaging (LSCI) (*n*=8), three independent experiments. Data presented as marginal means±s.e.m. **P<0.005, ***P<0.0005, determined by linear mixed modelling. PU, perfusion units. Representative LSCI images for placebo- and IL-1Ra-treated animals are shown. (E) Mice were subjected to collagenase-induced ICH and given IL-1Ra (*n*=6) or placebo (*n*=6) and culled 24 h later. Left: 20 µm coronal brain sections were immunostained for pan-laminin (cyan), cyclooxygenase-2 (magenta) and DAPI (white). One representative image from six animals per group shown. Scale bars: 50 μm. Right: cyclooxygenase-2 (COX-2)^+^ cells associated with vessels in the ipsilateral hemisphere were quantified. Data presented as mean±s.e.m. ****P<0.0001, determined by unpaired two-tailed *t*-test.

Having previously established that monocyte recruitment contributes to neuromotor injury and that IL-1 facilitates monocyte trafficking, we next looked to investigate whether IL-1 inhibition improved neuromotor outcome in our murine model. IL-1Ra treatment was given for 2 days (Fig. 5A) so that effects were limited to early detrimental monocyte recruitment and not the pro-reparative functions that are proposed to come with later phases of recruitment (Chang et al., 2018). IL-1Ra-treated animals performed worse on the rotarod assay within 24 h of ICH and did not improve over the 7 day testing period, with weight loss showing a similar trend (Fig. 5B; Fig. S5).

IL-1 is a pleiotropic cytokine that is proposed to sit at the apex of inflammation and coagulation. We therefore hypothesised that IL-1 inhibition worsened injury in our ICH model by preventing inflammation–coagulation cross-talk, resulting in larger bleed volumes. However, histological and biochemical analysis of blood load indicated that IL-1 inhibition did not affect bleed size (Fig. 5C).

IL-1 can also affect vascular tone, and has been shown to modulate both vasoconstriction and vasodilatation dependent on the signalling niche (Minghini et al., 1998; Murray et al., 2014; Osuka et al., 1997). To assess potential changes to CBF, mice underwent laser speckle contrast imaging 24 h post-haemorrhage. In line with others (Ke et al., 2012; Ropper and Zervas, 1982; Yang et al., 1994), a reduction in cortical CBF following striatal ICH was detected. The reduction in CBF was further exacerbated in mice treated with IL-1Ra (Fig. 5D). COX-2 (also known as MT-CO2) is a major downstream target of IL-1 that regulates vascular tone during inflammatory conditions by producing various eicosanoids. In line with this, there were ∼4-fold fewer COX-2-expressing cells within the cerebrovasculature of IL-1Ra-treated animals than in that of placebo-treated animals 24 h post-ICH (Fig. 5E).

## DISCUSSION

Despite being a major area of unmet clinical need, the pathophysiological consequences of ICH are poorly understood. Here, we present further evidence that ICH results in acute accumulation of detrimental myeloid cells within the brain. Using RNA-seq, histological analysis and *in vivo* perturbations, we identified IL-1 as an important contributor of myeloid cell recruitment. However, IL-1 was also required for efficient maintenance of CBF within the affected brain hemisphere, and the net effect of IL-1 inhibition worsened neuromotor dysfunction in this setting. Thus, we present evidence of dichotomous actions of IL-1 during ICH. It is important to note, however, that IL-1 inhibition began prior to ICH induction, to gain a holistic appreciation of IL-1 functions in the bleeding and clotting phases of ICH. Although these findings are mechanistically insightful, they are not predictive of therapeutic efficacy in delayed administration regimens.

Previous studies have shown that preventing Ly6C^hi^ monocyte infiltration improves motor function following ICH (Chang et al., 2018; Hammond et al., 2014a). Here we used *Ccr2*^−/−^ mice with known deficits in monocyte release from the bone marrow and recruitment to injured tissues owing to their inability to respond to the key monocyte chemotactic molecule CCL2 (Boring et al., 1997). Therefore, along with others, we have shown that monocytes contribute to injury during acute ICH and could thus represent a viable therapeutic target. However, T cells (Mack et al., 2001), endothelial cells (Dzenko et al., 2005) and border-associated macrophages (BAMs) (Van Hove et al., 2019) are also known to express CCR2 and may have a role. CCR2 on BAMs is linked to proliferation and migration in diseased brain states (Gómez-Nicola et al., 2014). We report CCR2 effects in the first 24 h of ICH, and this precedes brain microglia/macrophage proliferation (Taylor and Sansing, 2013). We observed an increase in the amount of T cells in the brain within this 24 h period; however, the adaptive immune system requires a longer period of activation than the innate immune system. It is, therefore, unlikely that either T cells or microglia/BAMs contribute to the CCR2-dependent effects seen here. Endothelial CCR2, on the other hand, is required for efficient monocyte transmigration and, thus, is likely to phenocopy monocyte CCR2 (Dzenko et al., 2005). Thus, the data presented here add to the growing evidence supporting an important role of monocytes in the pathophysiology of ICH (Chang et al., 2018; Hammond et al., 2014a,b; Yao and Tsirka, 2012). It is not currently clear how monocytes contribute to acute neurological injury in mouse models of ICH, and studies of other acute brain disease paradigms find opposing effects. In mouse models of IS, for example, monocytes have been shown to prevent haemorrhagic transformation by maintaining vascular integrity through TGF-β (Gliem et al., 2012), although this has been challenged more recently (Schmidt et al., 2017). The levels of CCL2 in ICH patient plasma correlate with disease severity, and one possibility is that monocytes contribute to haematoma expansion, as previous studies have shown delayed haematoma growth in *Ccr2*^−/−^ mice (Hammond et al., 2014a; Yao and Tsirka, 2012). However, monocytes enter the brain and, over time, transition to macrophages that are important for clearance of neurotoxic RBCs (Chang et al., 2018). It is therefore important to evaluate the long-term consequences of monocyte-targeting therapies in future studies.

We show that monocyte trafficking to the brain requires IL-1 following ICH. Tissue infiltration of myeloid cells requires transmigration through inflamed vasculature, and we identified IL-1-specific upregulation of the adhesion molecule VCAM1 that is essential for tethering of immune cells to the endothelium during inflammation. VCAM1 directly binds to the very-late antigen 4 complex that is part made up of the α4 integrin, and α4 inhibition has been previously shown to abrogate monocyte infiltration (Hammond et al., 2014b; Newham et al., 1997). It is probable, therefore, that IL-1 is sensed by the cerebrovasculature to promote brain trafficking of myeloid cells. Indeed, transgenic investigations indicate that brain endothelial-specific IL-1R1 facilitates myeloid cell trafficking to the brain and is responsible for neutrophil recruitment in a murine model of IS (Liu et al., 2019; Wong et al., 2019). In this study, we focused on the effects of IL-1 during the first 24 h of ICH, given evidence that inflammatory processes impact brain health during this time period (Lan et al., 2017; Mastorakos et al., 2021). We observed decreased myeloid cell trafficking (Fig. 4), rotarod performance and CBF (Fig. 5) at 24 h following IL-1Ra administration. However, we also observed sustained reduction in rotarod score that may be explained by different functions of IL-1 across the first 7 days of ICH. Given that the MNP response has been shown to move from detrimental to beneficial over time (Chang et al., 2018), and that IL-1 contributes to monocyte trafficking, the lack of temporal profiling of IL-1 signalling is an important limitation of this study.

Here, we showed that IL-1 produced by MNPs is responsible for a large proportion of myeloid cell trafficking to the haemorrhaged brain. An important next step is to determine the processes controlling infiltration of the residual myeloid cell population, which could be due to passive entry of these cells from the initial bleed, or by signalling through independent pathways such as TNF-α. Previous studies using post-mortem ICH patient tissue provided evidence of IL-1β production and pathway activation but did not identify the cell type responsible (Loan et al., 2022; Zhang et al., 2015). We show that MNPs, including microglia, produce IL-1β following clinical ICH, and these results confirm findings from perihaematomal resections without the confounding factor of surgical manipulation of the tissue (Li et al., 2017). Both IL-1α and IL-1β signal through IL-1R1, resulting in equivalent downstream signals. We found that IL-1α and IL-1β were produced by microglia within 4 h of collagenase-induced brain haemorrhage and that monocytes produced IL-1β for up to 24 h. The native 31 kDa pro-IL-1β molecule requires cleavage to adopt a form that can bind and activate IL-1R1. The best-studied IL-1β-processing enzyme is caspase-1, which is activated by multimolecular platforms termed inflammasomes (Schroder and Tschopp, 2010). There are a number of sensor molecules that trigger inflammasome activation upon recognition of DAMPs ranging from ATP to lysophosphotidylcholine (Broz and Dixit, 2016; Freeman et al., 2017). There is evidence the NLRP3 inflammasome contributes to immune cell trafficking following ICH (Ren et al., 2018). We previously showed that inflammasomes contribute to IS pathogenesis independently of NLRP3 (Denes et al., 2015; Lemarchand et al., 2019). Here, we adopted a strategy to block all inflammasomes in the acute phase of ICH by administering a caspase-1 inhibitor and found no effect on immune recruitment. Previous studies quantified inflammasome-dependent effects on myeloid cell trafficking 3 days post-injury, whereas we show no effect at 24 h. This temporal difference could have a large impact on findings as RBC lysis begins to release the NLRP3 inflammasome activator haem in the days post-ictus (Dutra et al., 2014). We did not measure caspase-1 activity in the brain following VX-765 treatment and thus cannot provide absolute evidence of caspase-1 inhibition within the brain. However, we used a dose that has shown efficacy in a mouse model of Alzheimer's disease (Flores et al., 2018), and we provide evidence that it did block inflammasome activation *in vitro*. Together, our results indicate that inflammasome activation is not required for immune cell trafficking during the first 24 h of ICH, although caution is advised given the limitation that caspase-1 inhibition was not validated *in vivo*.

We also showed accumulation of CD11b^+^ and IL-1β^+^ cells in regions of intraparenchymal haemorrhage in a human cSVD cohort. Intraparenchymal haemorrhages were rare in this cohort (2/36), and we cannot determine the chronicity of the responses in these cases. These findings highlight a need for better profiling of the molecular, cellular and temporal pathophysiology of cSVD-related brain haemorrhages to better understand conserved processes between acute and chronic conditions.

ICH was first thought to be accompanied by a perihaematomal ischaemic region similar to the IS penumbra. Many studies had identified regions of hypoperfusion in both clinical and preclinical investigations (Fainardi et al., 2008; Ke et al., 2012; Mayer et al., 1998; Nath et al., 1987; Ropper and Zervas, 1982; Tayal et al., 2007; Yang et al., 1994). However, quantification of metabolic function in the hypoperfused region, using the haemodynamic readout of oxygen extraction fraction, could not provide evidence of ischaemia (Zazulia et al., 2001). There is evidence that quantitative changes in CBF can predict outcome, however (Tayal et al., 2007; Xu et al., 2013). Owing to the brain’s inability to store energy, the cerebrovasculature has evolved multi-level mechanisms to tightly control the supply of blood to the organ. Disruption to this supply of blood in healthy individuals can have profound impacts on cognitive function (Marshall, 2001), and pathological reduction of CBF is linked to brain atrophy in clinical (Arba et al., 2017) and preclinical (Nishio et al., 2010) settings, all in the absence of ischaemia. In agreement with others, we observed reductions in cortical CBF following striatal ICH (Ke et al., 2012; Ropper and Zervas, 1982; Yang et al., 1994). We discovered that IL-1-dependent processes increase CBF following ICH, and this correlates with reduced neuromotor injury. Thus, we propose a model whereby an inflammatory reaction to ICH forms part of a reactive vasodilatory response. Accordingly, previous preclinical studies have noted transient reductions in CBF that normalise once the immune response heightens (Ahn et al., 2018; Rosidi et al., 2011).

We hypothesise that the effect of IL-1 on CBF could be via its common downstream target COX-2. Indeed, longitudinal profiling of haematomal myeloid cells has recently shown that prostaglandin E2 (PGE_2_) levels correlate with better clinical outcome (Askenase et al., 2021). The five prostaglandins produced via COX-2 can act on a range of receptors, leading to spatial- and context-specific effects. PGE_2_, for example, constricts vessels when acting on EP_1/3_ (also known as PTGER1/3) receptors yet dilates vessels when acting on EP_2/4_ (also known as PTGER2/4) receptors (Yang and Du, 2012). IL-1 itself has also been shown to have contrasting effects on blood flow. Application of IL-1 into musculoskeletal arteries *in vivo* causes vasodilation, whereas isolation and stimulation of the same arteries *ex vivo* has no effect (Minghini et al., 1998). IL-1 coupled to acute cerebral ischaemia causes endothelin-1 release, leading to reduced haemodynamics (Murray et al., 2014); however, injections of IL-1 into the basilar artery lead to prostacyclin production and subsequent vasodilation (Osuka et al., 1997). The vast majority of vascular mural cells express IL-1R1, and the complex actions of IL-1 on vascular tone are likely due to cell-specific effects. Further studies are therefore needed to delineate the cerebral cell-specific actions of IL-1 and COX-2 during steady state and disease. To do this, transgenic mouse lines could be developed to restrict IL-1R1 expression to discrete cell types, similarly to those described by Liu et al. (2019).

The dichotomous functions of IL-1 observed in this study could also be explained by functional differences between the ligands. As discussed earlier, activated IL-1β is released following caspase-1 activation. IL-1α, on the other hand, can be released during cellular necrosis and become activated by extracellular proteases, such as the ICH-relevant thrombin (Burzynski et al., 2019). There is also evidence that IL-1α localises and functions in the nucleus and at the cell membrane (Mantovani et al., 2019; Wellens et al., 2024), collectively suggesting that the functions of IL-1 ligands are contextually and environmentally specific. IL-1 signalling was inhibited at the shared receptor in this study. Therefore, delineating differential functions of the ligands is an important area of future study that may separate protective functions from damaging ones, as recently shown for IL-1α in IS (Lemarchand et al., 2025). However, it must be noted that IL-1α and IL-1β have also been reported to have a degree of redundancy (Boutin et al., 2001).

There is now considerable evidence that delayed administration of IL-1Ra improves outcome in preclinical models of IS (Sobowale et al., 2016). IL-1Ra treatment for IS patients has also shown promise in trials conducted prior to the uptake of thrombolytic therapy. However, in a more recent clinical trial, IL-1Ra was postulated to potentially have negative interactions with thrombolysis (Smith et al., 2018). In contrast to all the clinical studies of IL-1Ra (Emsley et al., 2005; Smith et al., 2018; Parry-Jones et al., 2023; Singh et al., 2014), here, we injected IL-1Ra before the onset of ICH to uncover details of IL-1-dependent neuroimmune processes and found surprising negative impacts on neuromotor function. The degradation of intravascular clots through thrombolysis may release factors similar to those found in a bleeding brain. Consequently, we hypothesise that haemostatic environments lacking the buffer of IL-1-induced prostaglandins have vascular tone skewed toward vasoconstriction through the large amounts of thromboxane A_2_ released from platelets. ICH was induced in deep-brain regions in this paper, so it is currently unclear how well our findings generalise to lobar ICH. Thus, important next steps are to (1) define interactions between IL-1 and haemostatic processes, and (2) test the effects of delayed administration of IL-1Ra in preclinical models of both lobar and deep ICH.

## MATERIALS AND METHODS

### Immunohistochemistry/immunofluorescence labelling of post-mortem human brain tissue

cSVD and ICH brain tissue samples were acquired from the South West and Edinburgh arms of the Medical Research Council Brain Banks, respectively. Samples were taken from basal ganglia of two males and one female (age range 63-86 years) who died within 3 days of ICH, three females (age range 89-95 years) with a clinical diagnosis of dementia, histopathological evidence of cSVD and extravascular RBCs, and three matched (basal ganglia) controls of two males and one female (age range 63-79 years) from the Lothian Birth Cohort (Deary et al., 2012). cSVD cases were triaged to identify those with evidence of recent brain haemorrhage (containing intact RBCs) in the basal ganglia (3/36, 8%), and these three cases were selected, although only two of these were found to be parenchymal upon immunostaining. Brain sections were deparaffinised and rehydrated prior to undergoing relevant heat-mediated antigen retrieval in a water bath set to 97.5°C for 30 min. Antigen retrieval was performed using sodium-citrate pH 6.0 solution for rabbit-anti-CD11b (0.68-6.75 µg ml^−1^, EPR1344, ab209970, Abcam, RRID:AB_2915959) or Tris-EDTA pH 9.0 solution for rabbit-anti-Iba1 (2.56 µg ml^−1^, EPR16588, ab178846, Abcam, RRID:AB_2636859) and goat-anti-IL-1β (2.00 µg ml^−1^, AF-201, R&D Systems, RRID:AB_354387). Primary antibodies were incubated overnight at 4°C in primary antibody buffer [1% bovine serum albumin (BSA), 0.3% Triton X-100, 0.05% sodium azide, PBS], followed by incubation with secondary antibodies for 90 min at room temperature (RT) in secondary antibody buffer (0.1% BSA in Tris-buffered saline). The following secondary antibodies were used: donkey-anti-rabbit Alexa Fluor^®^ 647 (10 µg ml^−1^, Abcam) and donkey-anti-rat Alexa Fluor^®^ 647 (10 µg ml^−1^, Abcam). In order to amplify low-abundance epitopes or overcome RBC autofluorescence, the Tyramide SuperBoostTM (B40936, Thermo Fisher Scientific) protocol was followed as per the manufacturer's instructions using biotinylated horse-anti-goat IgG (7.5 µg ml^−1^, BA-9500, Vector Laboratories) or goat-anti-rat IgG (7.5 µg ml^−1^, BA-9400, Vector Laboratories) secondary antibodies. Wash steps were performed throughout using wash buffer (0.1% Tween-20 in Tris-buffered saline).

### Animals

Animal procedures were carried out in accordance with the Animal Scientific Procedures Act (1986) and the European Council Directive 2010/63/EU, and were approved by the Animal Welfare and Ethical Review Board, The University of Manchester, UK, and the Animal Care and Use Committee of the Institute of Experimental Medicine, Budapest, Hungary. For behavioural analyses, sample size was calculated by power analysis using a significance level of α=0.05 with 80% power to detect statistical differences. For other analyses such as immunohistochemistry, sample size was chosen based on prior knowledge of the techniques. *Ccr2*^−/−^ animals [originally from The Jackson Laboratory (Boring et al., 1997)] were bred in-house and shared by J. Grainger (The University of Manchester, Manchester, UK), and were backcrossed to a C57BL/6 background for at least ten generations. For microglia depletion experiments, mice were bred at the specific pathogen-free unit of the Animal Care Unit of the Institute of Experimental Medicine (IEM HAS, Budapest, Hungary). Mice had free access to food and water and were housed under light-, humidity- and temperature-controlled conditions. Experiments followed Animal Research: Reporting of *In Vivo* Experiments (ARRIVE) guidelines (Kilkenny et al., 2013). Animals were maintained under standard laboratory conditions: ambient temperatures of 21°C (±2°C), humidity of 40-50%, 12 h light cycle, *ad libitum* access to water and standard rodent chow. All surgeries were performed with the surgeon concealed to the treatment and/or genotype, and all behavioural and histological analyses were performed by an observer unaware of genotype/condition. Treatments were randomly allocated using the research randomiser tool. 4- to 6-month-old male mice were used.

### Surgery

A model of intraparenchymal brain haemorrhage was used that created an active bleed within the brain by using collagenase VII-S (C2399, Sigma-Aldrich) to break down the basal lamina of cerebrovascular beds (Kirkman et al., 2011). To do this, mice were initially induced under anaesthesia using 4% isoflurane in 70% N_2_O and 30% O_2_, and fur was shaven from the head. Mice were then transferred to a feedback-controlled heating pad set to 37°C, securely fixed to a stereotactic frame (Stoetling), anaesthesia was maintained at 1.5-2.0% isoflurane in 30% N_2_O and 70% O_2_ using a nose cone, and Videne (EcoLab) was applied to the scalp. A longitudinal midline incision above the skull was performed, and periosteum was stripped away from the midline on both sides. A burr hole was created using a micro-drill and a glass micropipette (BLAUBRAND, pulled at 70°C) inserted at the following co-ordinates from bregma: anterior–posterior, 0.0 mm; lateral, −2.0 mm; deep, −2.7 mm. 0.5 µl of 0.09 units µl^−1^ of collagenase dissolved in saline was then injected at a rate of 1 µl min^−1^. The needle was left *in situ* for 10 min before removal, and wounds were sutured. The scalp was cleaned once more with Videne, and a topical analgesia was applied (EMLA cream, AstraZeneca) before animals were given a subcutaneous bolus of saline (10 ml kg^−1^) and buprenorphine (50 µg kg^−1^, Vetergesic, CEVA Animal Health Ltd, High Wycombe, UK) before being recovered in 28°C housing. Animals were then transferred to ventilated cages suspended over a heating pad with free access to mashed food and water in normal housing conditions.

The 0.5 μl injection volume limits the amount of solution leaking from the brain following injection. The 0.09 units µl^−1^ dose was optimised in-house to produce ICH with limited animal suffering while retaining measurable neuromotor deficits. Mice with successful ICH show unilateral movement when suspended by the tail at 24 h. Failure to show this deficit was a pre-defined exclusion criterion.

### Microglia depletion

PLX5622 was provided by Plexxicon Inc. (Berkely, CA, USA) and formulated by Research Diets (New Brunswick, NJ, USA) into an AIN-76A standard chow in 1200 ppm (1200 mg PLX5622 in 1 kg chow). Mice were fed PLX5622 for 14 days to eliminate microglia from the brain. No sign of physiological illness (alterations in food intake, weight or physical appearance) or overt behavioural changes (social interactions, exploration) were observed during the diet period, in accordance with other studies (Fekete et al., 2018).

### IL-1R1 inhibition

Human IL-1Ra (Kineret, SOBI) was supplied (in 10 mM sodium citrate, 140 mM NaCl, 4 µM EDTA, 5.34 µM polysorbate 80, pH 6.5) from the hospital pharmacy and further diluted in sterile saline. Placebo controls contained vehicle only. We injected 10 µg of the naturally occurring antagonist IL-1Ra intrastriatally (bregma: anterior–posterior, 0.0 mm; lateral, −2.0 mm; deep, −2.7 mm) 10 min prior to collagenase injection, followed by a 100 mg kg^−1^ subcutaneous dose. In order to maintain adequate plasma concentrations of IL-1Ra, a second subcutaneous 100 mg kg^−1^ dose was injected 6 h later (Greenhalgh et al., 2010). Where animals were kept alive for more than 24 h, 100 mg kg^−1^ doses were given subcutaneously in the morning and evening each day ending in the evening of the second day following surgery. IL-1Ra was well tolerated, with mice showing no overt signs of physiological illness.

### Caspase-1 inhibition

To inhibit caspase-1 during brain haemorrhage, we used the caspase-1 inhibitor VX-765 (S2228, Selleckchem) that has recently been shown to be efficacious in mouse models of Alzheimer's disease (Flores et al., 2018). We adopted the same dosing regimen used in our IL-1Ra model with a single 7.5 µg intrastriatal injection followed by 50 mg kg^−1^ subcutaneous doses. VX-765 was diluted in a sterile 5% Cremophor EL (Sigma-Aldrich), 5% DMSO (Sigma-Aldrich), 90% saline mixture. Placebo contained vehicle alone. VX-765 was well tolerated, with mice showing no overt signs of physiological illness.

### Rotarod assay

Mice were allowed to habituate to the behavioural room and handled for at least 1 week prior to commencement of training. Animals were trained for 3 consecutive days prior to surgery using the following protocol. The rotarod was set to loading phase while animals were placed onto the rotarod. Following a 15 s loading period, the rotarod was set to accelerate from 4 to 40 rpm over 300 s. An animal was deemed to have completed the task when it had fallen from the apparatus, accrued three passive rotations or stayed on the apparatus until 300 s. The time at which an animal completed the task was recorded. Once all animals had finished the run, the apparatus was thoroughly cleaned with a 70% ethanol solution. Three runs were performed per day with a 15 min inter-trial interval. On the third and final day of training, the results from the final two runs were averaged and set as the baseline run time for that animal. On test days following ICH, the same protocol was followed, with animals performing three runs and the times from the final two runs averaged for the animals score.

### Laser speckle contrast imaging

Mice were anaesthetised and placed in a stereotaxic frame (World Precision Instruments, Sarasota, FL, USA) positioned under a moor FLPI2 Full-Field Perfusion Imager (Moor Instruments, Axminster, UK). Anaesthesia was maintained at 1.5% isoflurane, and body temperature was maintained at 37°C using a feedback-controlled heating pad. A longitudinal midline incision was performed on the scalp to expose the skull. The skull was cleared of any hair, and acoustic gel was applied before a glass coverslip was mounted atop. Imaging was conducted for 3 min with a 4 s per image acquisition rate, 20 ms exposure time and 100 frame filter. Mice were imaged 24 h post-haemorrhage, and regions of interest were drawn around each hemisphere using moor FLPI2 Full-Field Laser Perfusion Imager Review V5.0 software. Flux values were averaged across all timepoints and taken for further analysis.

### Tissue processing and immunostaining

Mice were transcardially perfused with PBS followed by 4% paraformaldehyde (PFA) under isoflurane or ketamine-xylazine induced terminal anaesthesia. Brains were removed and post-fixed in 4% PFA for 24 h at 4°C before being cryoprotected in a 20% sucrose solution at 4°C for up to 4 days. Brains were then frozen in isopentane (−50°C to −60°C), and 20 µm coronal sections were taken every 400 µm using a Leica cryostat. For immunostaining, brain sections were dried for 24 h prior to undergoing relevant heat-mediated antigen retrieval in a water bath set to 97.5°C. Antigen retrieval was performed using Tris-EDTA pH 8.6 solution for 20 min for the following antibodies: rabbit-anti-Iba1 (2.56 µg ml^−1^, EPR16588, ab178846, Abcam, RRID:AB_2636859), goat-anti-IL-1α (1 µg ml^−1^, AF-400, R&D Systems, RRID:AB_354473), goat-anti-IL-1β (1 µg ml^−1^, AF-401, R&D Systems, RRID:AB_416684), rat-anti-Ly6G (1.25 µg ml^−1^, 1A8, BioLegend, RRID:AB_1089179), rabbit-anti-collagen IV (5 µg ml^−1^, ab19808, Abcam, RRID:AB_445160), goat-anti-vascular cell adhesion protein 1 (2 µg ml^−1^, AF643, R&D Systems, RRID:AB_355499), goat-anti-cyclooxygenase-2 (0.5 µg ml^−1^, AF4198, R&D Systems, RRID:AB_2229909) and rabbit-anti-laminin (0.25 µg ml^−1^, ab11575, Abcam, RRID:AB_298179). Antigen retrieval was performed using sodium citrate pH 6.0 solution for rabbit-anti-CD11b (2.70 µg ml^−1^, EPR1344, ab209970, Abcam, RRID:AB_2915959). Primary antibodies were incubated overnight at 4°C in primary antibody buffer (1% BSA, 0.3% Triton-X-100, 0.05% sodium azide, PBS), followed by incubation with secondary antibodies for 90 min at RT in secondary antibody buffer (0.1% BSA in Tris-buffered saline). The following secondary antibodies were used: donkey-anti-rabbit Alexa Fluor^®^ 647 (10 µg ml^−1^, Abcam), donkey-anti-rat Alexa Fluor^®^ 647 (10 µg ml^−1^, Abcam). In order to amplify low abundance epitopes or overcome RBC autofluorescence, the Tyramide SuperBoostTM (B40936, Thermo Fisher Scientific) protocol was followed as per manufacturer instructions using biotinylated horse-anti-goat IgG (7.5 µg ml^−1^, BA-9500, Vector Laboratories), goat-anti-rat IgG (7.5 µg ml^−1^, BA-9400, Vector Laboratories) or goat-anti-rabbit IgG (7.5 µg ml^−1^, BA-1000, Vector Laboratories) secondary antibodies. Wash steps were performed throughout using wash buffer (0.1% Tween-20 in Tris-buffered saline). RBCs were visualised by autofluorescence in the 488 channel following antigen retrieval.

### Microscopy

Images were collected on a Zeiss Axioimager.D2 or Olympus BX63 upright microscope and captured using a Coolsnap HQ2 camera (Photometrics) or DP80 camera (Olympus) through Micromanager software v1.4.23 or CellSens Dimensions V1.16 (Olympus). Specific band pass filter sets were used to prevent bleed through from one channel to the next. Images were then processed and analysed using Fiji ImageJ. Four regions of interest that spanned haematoma and perihaematoma were taken across four different brain sections for histological quantification.

### Haematoma analysis

For histological quantification of haematoma volume, brains were sectioned at 400 µm intervals and digitally scanned, and regions of interest (ROI) were drawn around the haematoma on each section using ImageJ. Haematoma volume was calculated by summing the area of all ROIs and multiplying by the inter-section interval (400 µm). For biochemical quantification of blood load, perfused brains were snap frozen before each hemisphere was isolated and lysed on ice in NP-40 lysis buffer containing protease inhibitor cocktail (PIC; 539131, Merck) using a handheld homogeniser. Lysates were centrifuged at 18,000 ***g*** for 10 min at 4°C, and supernatants were collected and ultracentrifuged at 100,000 ***g*** for 30 min at 4°C. Haemoglobin content of S100 fractions were quantified using a haemoglobin assay kit (MAK115-1KT, Sigma-Aldrich) as per the manufacturer's protocol with slight modification to reduce assay volume by 50%. Samples were run in triplicate at a range of dilution factors, and values closest to the standard were used in analysis. Both haematoma quantification experiments were performed by an operator unaware of treatment.

### Cell isolation

Mice were transcardially perfused with PBS, and ipsilateral hemispheres were digested for 60 min at 37°C with 50 U ml^−1^ collagenase (17104-019, Gibco), 0.5 U ml^−1^ Dispase II (17105-041, Gibco) and 200 U ml^−1^ DNase I (10104159001, Roche) in Hank's balanced-salt solution containing calcium and magnesium. The tissue was then mechanically dissociated using a dounce homogeniser, and myelin was removed using a 32% isotonic percoll solution. Then, the solution was centrifuged at 2000 ***g*** for 10 min at 4°C with brake on 2. When possible, cells were kept in ice-cold RPMI containing 3% foetal bovine serum (FBS; Life Technologies). Following single-cell isolation, RBCs were lysed by 3 min incubation with BD Pharmlyse (555899, BD Bioscience).

### Western blotting

Following single-cell isolation, myeloid cells were purified using CD11b^+^ magnabeads (130-093-636, Miltenyi) according to the manufacturer's protocol. Positive and negative populations were captured and lysed in NP-40 lysis buffer (150 mM NaCl, 50 mM Tris-HCl, 1% NP-40, pH 7.4) supplemented with PIC (539131, Merck). Lysates were run on 12% polyacrylamide gels and transferred onto nitrocellulose membranes using the mixed molecular mass setting of a Trans-Blot Turbo system (Bio-Rad). Membranes were blocked in 5% BSA PBS containing 0.1% Tween-20 (Sigma-Aldrich) (PBST) for 1 h at room temperature. Following blocking, membranes were incubated (4°C) overnight with goat-anti-mouse IL-1β (100 ng ml^−1^, R&D Systems) or goat-anti-mouse IL-1α (100 ng ml^−1^, R&D Systems) in PBST containing 0.1% BSA. Membranes were then washed and incubated (room temperature) with rabbit anti-goat (550 ng ml^−1^, DAKO) in 0.1% BSA PBST. After washing, membranes were incubated in Amersham ECL Western Blotting Detection Reagent (GE Life Sciences) before detection using a G:BOX Chemi XX6 imaging system (Syngene).

### Flow cytometry

Isolated single-cell pellets were resuspended in FACS staining buffer (PBS with 2 mM EDTA) containing a number of the following antibodies: 50 µg ml^−1^ FC block (Clone 93, ebioscience, RRID:AB_312801), 1:500 Live/Dead Blue (Thermo Fisher Scientific), 0.4 µg ml^−1^ CD11b-BV786 (Clone M1/70, BD Bioscience, RRID:AB_3685114), 2 µg ml^−1^ CD3-BV510 (Clone 17A2, BioLegend, RRID:AB_2561387), 0.67 µg ml^−1^ CX3CR1-PE/Cy7 (Clone SA011F11, BioLegend, RRID:AB_2565700), 0.67 µg ml^−1^ CD45-PerCP/Cy5.5 (Clone 30-F11, eBioscience, RRID:AB_394003), 2 µg ml^−1^ IL-1β-PE (Clone NJTEN3, Thermo Fisher Scientific, RRID:AB_10732630), 1 µg ml^−1^ Ly6C-FITC (Clone AL-21, BD Bioscience, RRID:AB_394628), 0.67 µg ml^−1^ CD45r-APC/Cy7 (Clone RA3-6B2, BioLegend, RRID:AB_313007) and 1 µg ml^−1^ Ly6G-APC (Clone 1A8, BD Bioscience, RRID:AB_1727561). 2 µg ml^−1^ Rat/IgG1,kappa-PE (Thermo Fisher Scientific) was used as isotype control for IL-1β, and fluorescence minus one controls were used to set gates for all other stains. Cell numbers were quantified using CountBright™ counting beads (C36950, Thermo Fisher Scientific). Samples were acquired on a BD LSR II (Becton Dickenson) using BD FACSDiva software (Becton Dickenson). Data were analysed using FlowJo V.10 (Tree Star Inc.).

### RNA isolation

Mice were PBS perfused, and RNA was isolated from the right hemisphere (ipsilateral in haemorrhaged animals) using an RNeasy lipid tissue mini kit (Qiagen) as per the manufacturer's protocol. Brain hemispheres were homogenised using lysing matrix D tubes (MPBio) in a Qiagen Tissue Lyser II.

### RNA-seq

RNA-seq analysis was performed. RNA samples were assessed for quality and integrity using a 2200 TapeStation (Agilent Technologies) according to the manufacturer's instructions. RNA-seq libraries were generated using TruSeq Stranded mRNA assay (Illumina) according to the manufacturer's instructions. Briefly, poly-T, oligo-attached, magnetic beads were used to extract polyadenylated mRNA from 1 μg total RNA. The mRNA was then fragmented using divalent cations under high temperature and transcribed into first-strand cDNA using random primers. Second-strand cDNA was then synthesised using DNA polymerase I and RNase H, and a single ‘A’ base addition was performed. Adapters were then ligated to the cDNA fragments and purified and enriched by PCR to create the final cDNA library. Adapter indices were used to multiplex libraries, which were pooled prior to cluster generation using a cBot instrument. The loaded flow cell was then pair-end sequenced (101+101 cycles, plus indices) on an Illumina HiSeq4000 instrument. Demultiplexing of the output data (allowing one mismatch) and BCL-to-Fastq conversion was performed with CASAVA 1.8.3. Sequencing quality for each sample was determined using the FastQC program. Low-quality sequence data were removed utilising the trimmomatic program. STAR v2.4.0 was utilised to map the trimmed sequence into the murine genome (mm10 genome with gencode M16 annotation). Raw counts for each sample were generated by the htseq-count program and subsequently normalised relative to respective library sizes using DESeq2 package for the R statistical program. The DESeq2 program was additionally used to plot the principal component analysis (PCA) with all sample data to visualise different clusters at multiple levels that describes the maximum variance within the dataset. Log2 fold change (log2FC) values were shrunken using the apeglm estimator. Genes of interest were identified by pairwise comparisons. False discovery rate (FDR)-adjusted *P*-values were used to evaluate significance.

### Comparison with previous studies

Data were downloaded and filtered for significantly enriched terms [logFC>1 (Carmichael et al., 2008), log2FC>0 (Rosell (Rosell et al., 2011)] before intersecting genes across all datasets. The ggvenn R package was utilised to create a Venn diagram visualising overlapping features. The 23 genes found enriched across all studies were entered into the STRING platform and clustered using the MCL algorithm with inflation parameter set to 3. Functional enrichment analysis of the 23 genes was performed against Gene Ontology Biological Processes terms using the gProfiler2 R package.

### Functional and pathway enrichment analysis

Genes with an FDR-corrected *P*-value of less than 0.01 were analysed for transcriptional regulation and cell pathway enrichment utilising the enrichR package (Jawaid, 2019) to string search the Enrichr webserver, GO term, PANTHER and Transcriptional Regulatory Relationships Unraveled by Sentence-based Text mining (Han et al., 2018) datasets (Chen et al., 2013; Kuleshov et al., 2016) on R. Significant features (*P*<0.05) were further probed by cluster analyses utilising Ward's minimum variance method of hierarchical clustering visualised using R package ‘pheatmaps’. Heatmap, PCA and volcano plots were generated using ‘pheatmaps’, R version 2.6.1 base and ‘ggplot2’ (Bodenhofer et al., 2011), respectively. GSEA was performed using the GSEA software (MSigDB).

### Cell culture

To generate primary mouse bone marrow-derived macrophage cultures, bone marrow from wild-type (C57BL/6) mice were isolated and cultured for 7 days with 30% L929 mouse fibroblast supernatant-conditioned Dulbecco's modified Eagle medium containing 10% FBS (Life Technologies), 100 U ml^−1^ penicillin and 100 μg ml^−1^ streptomycin.

### Statistical analyses

Data are presented as means+s.e.m. apart from laser speckle contrast imaging (LSCI) data, which are presented as marginal means±s.e. For LSCI and behavioural analyses, linear mixed modelling was used to evaluate the effect of independent factors (treatment and time/hemisphere) on the dependent variable. All factors and interactions were modelled as fixed effects. A within-subject design with random intercepts was used for all models. The significance of inclusion of a dependent variable or interaction terms was evaluated using log-likelihood ratio. Homoscedasticity and normality were evaluated graphically using predicted versus residual and Q-Q plots, respectively. All analyses were performed using R (version 3.3.3). Statistical analyses for everything else were performed using unpaired two-tailed *t*-tests, one-way ANOVA and two-way ANOVA with Sidak corrected post hoc. Equal variance and normality were assessed with the Levene's test and the Shapiro–Wilk test, respectively, and appropriate transformations were applied when necessary. *P*<0.05 was considered significant. The latter statistical analyses were carried out using GraphPad Prism. Images were processed using Fiji ImageJ49 and analysed by manual counting with an experimenter unaware of image identity throughout. Flow cytometry data were analysed and populations were quantified using FlowJo V10.

## Supplementary Material

10.1242/dmm.052306_sup1 Supplementary information
