# Supplementary material for: Interleukin-1 regulates myeloid cell trafficking and cerebral blood flow following intracerebral haemorrhage
Source: Dis Model Mech. 2025 Sep 30;18(10):dmm052306. doi: 10.1242/dmm.052306 (PMC12519545; doi:10.1242/dmm.052306)
Supplement: Supplementary information [file dmm-18-052306-s1.pdf]

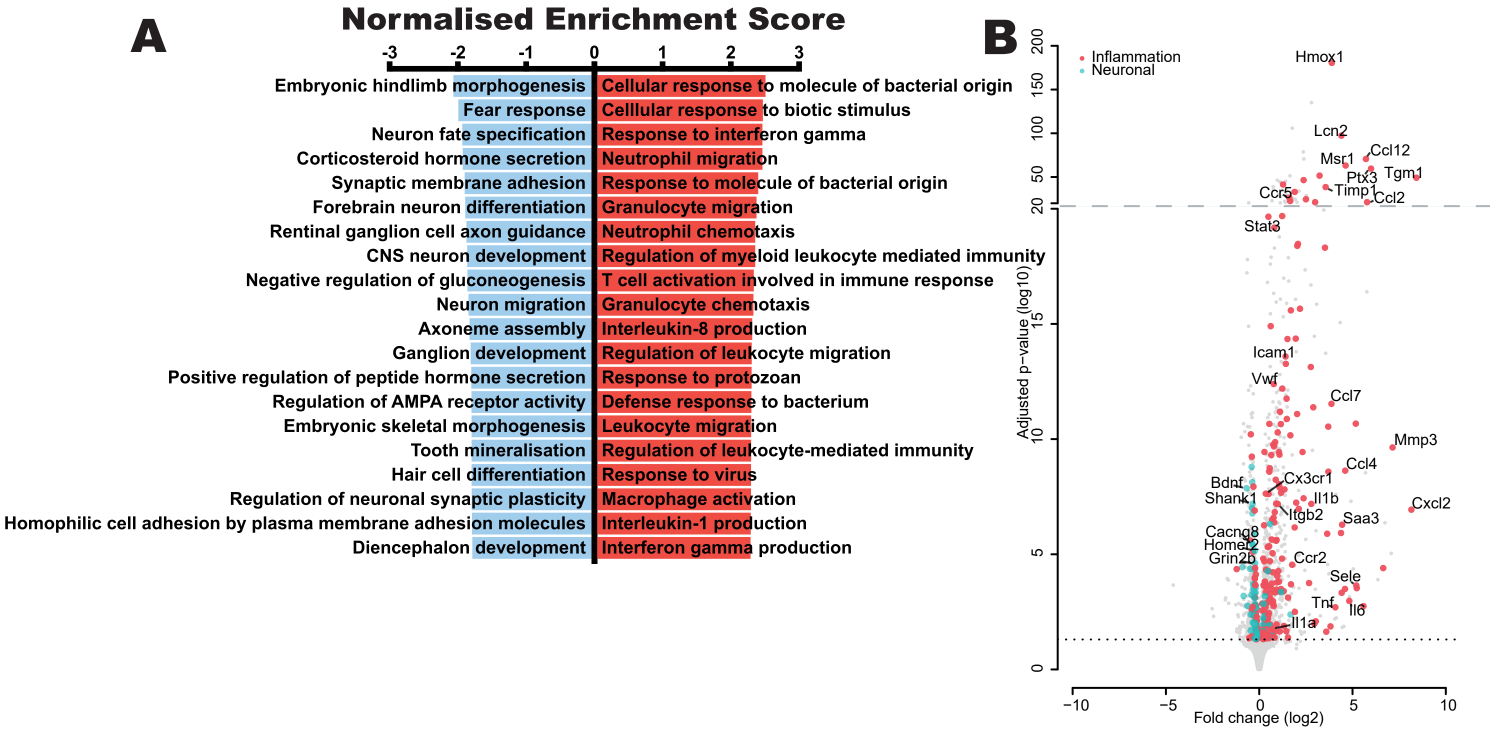

Fig. S1. RNAseq analysis related to Figure 1. (A) Top 20 negatively and positively significantly enriched gene sets in haemorrhaged brains based on GSEA. (B) Volcano plot highlighting genes belonging to inflammation (red) and neuronal (turquoise) pathways.

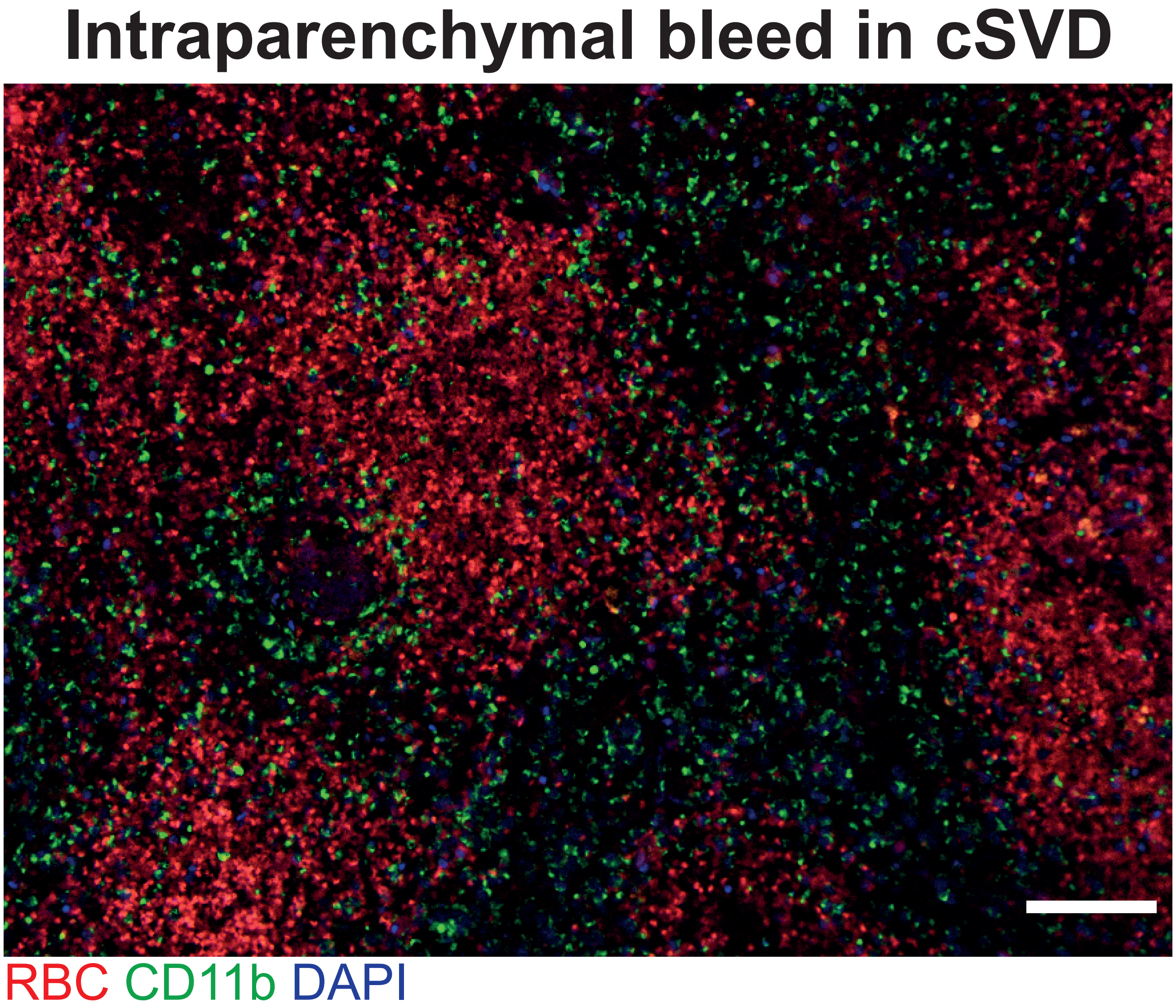

Fig. S2. Accumulation of myeloid cells in regions of heamorrhage pathology in human cerebral small vessel disease. Formalin-fixed paraffin-embedded post-mortem human brain tissue from a cerebral small vessel disease case was immunostained for the myeloid cell marker CD11b (green), DAPI (blue) and red blood cell autofluorescence (RBC) can be seen in red, scale bar = 100 μm.

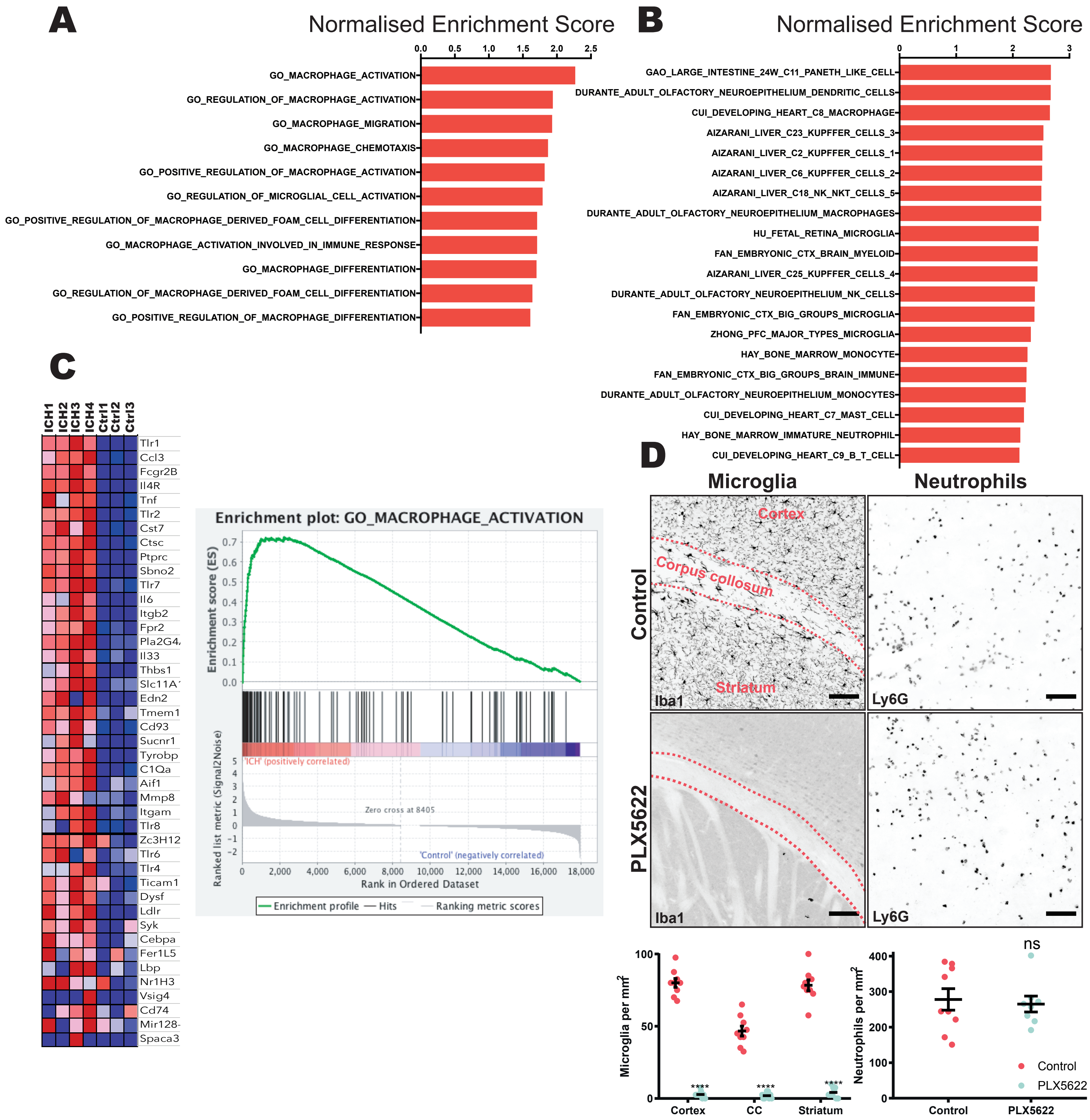

Fig. S3. Changes in macrophage-related genes predominate after ICH. (A) Macrophage activity related gene sets significantly enriched. (B) Gene sets related to macrophage subsets enriched. (C) Heatmap and enrichment plot of macrophage activation genes enriched in intracerebral haemorrhage. (F) Mice were fed a control (*n* = 10) or PLX5622 (CSF1R antagonist) (*n* = 10) containing diet for 14 days prior to collagenase injection and Iba1 was used to quantify microglia in the cortex, corpus collosum (CC) and striatum of brain sections. Haematomal neutrophils were quantified using Ly6G. One representative image from each group shown with scale bar 50 µm. Data presented as mean ± s.e.m, ns = not significant; **** = P<0.0001, determined by mixed-effects model with Sidak's post-hoc test.

Disease Models & Mechanisms • Supplementary information

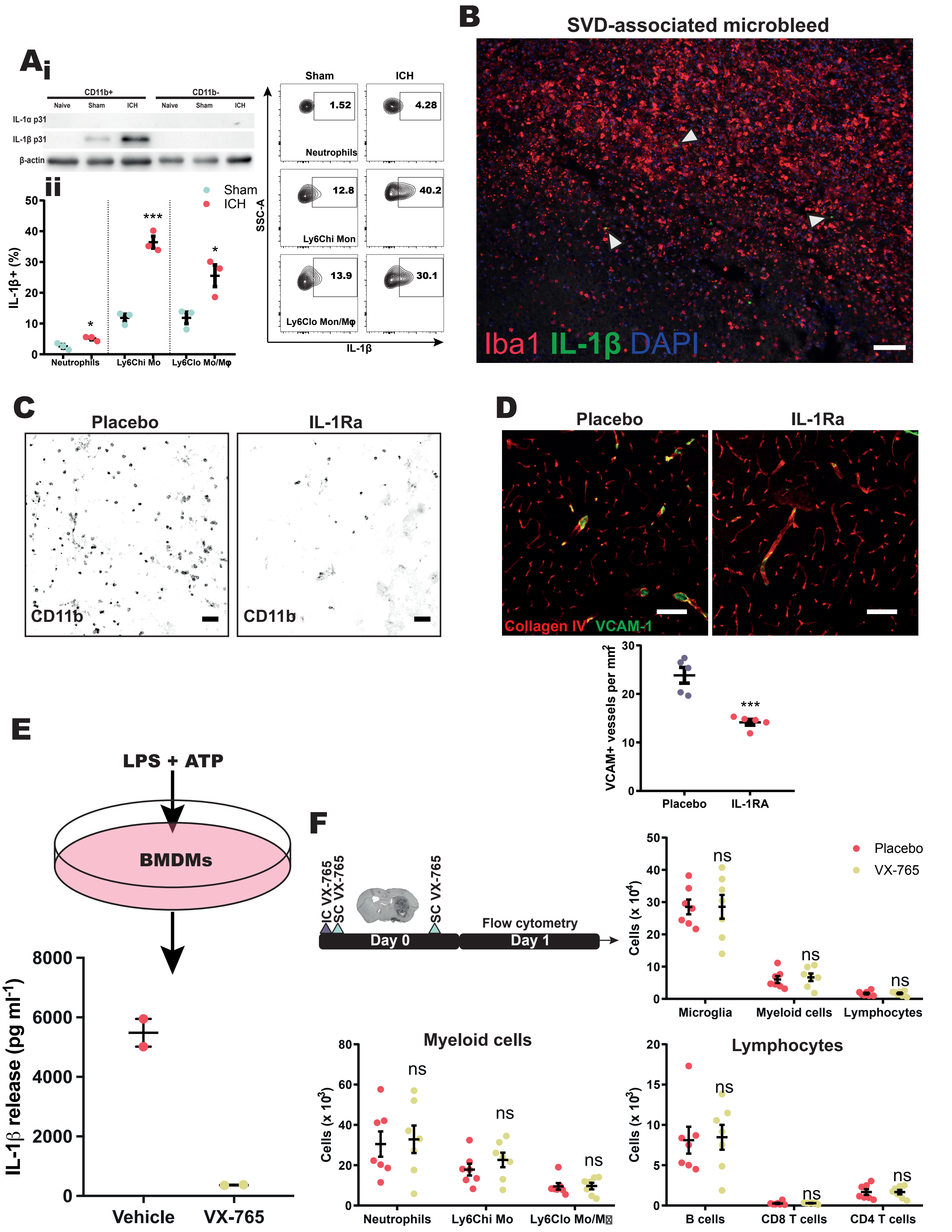

Fig. S4. Changes in macrophage-related genes predominate after ICH. (A) Single cells were isolated from the right hemisphere of naive, sham-operated, and collagenase-induced intracerebral haemorrhage (ICH) mice 24h post-surgery, CD11b+ cells were purified by magnabead separation and CD11b+ and CD11b- lysates immunoblotted for pro-IL-1α (31 kDa), pro-IL-1β (31 kDa) and β-actin (42 kDa) (i). (Aii) Flow cytometric analysis of IL-1β+ myeloid cells in the brains of sham-operated and ICH mice 24 h post-surgery. *n* = 3. Data presented as mean + s.e.m, *n* = 6. (B) Brain tissue from cSVD patients with intraparenchymal haemorrhage were immunostained for Iba1 (red), IL-1β (green) and DAPI (blue). Red blood cell (RBC) autofluorescence can be seen in white. One representative image from 2 patients is shown; scale bar 100 μm. Arrows indicate IL-1β+ cells. (C - D) Mice were treated with IL-1RA or placebo and subjected to ICH then culled at 24 h. 20 μm coronal brain sections were immunostained for CD11b (C) and collagen IV (red) and VCAM-1 (green) (D). One representative image from 8 biological repeats shown; scale bar 50 μm. (E) Primary murine bone marrow derived macrophages were treated with LPS (4 h) then incubated with 1.5 μM VX-765 or vehicle (15 min) and NLRP3 activated with 5 mM ATP (1 h). IL-1β release was measured by ELISA on supernatants (*n* = 2) (F) Mice were intrastriatally and subcutaneously injected with 7.5 μg and 50 mg kg-1 of the caspase-1 inhibitor VX-765, respectively, or placebo, prior to collagenase injection, followed by a second subcutaneous 50 mg kg-1 dose 6 hours later (*n* = 7), three independent experiments. Single cells were isolated 24h post-surgery and immunophenotyped using flow cytometry. Data presented as mean + s.e.m, ns = not significant; * = P<0.05; ** = P<0.01; *** = P<0.001; **** = P<0.0001, determined by unpaired two-tailed t-test.

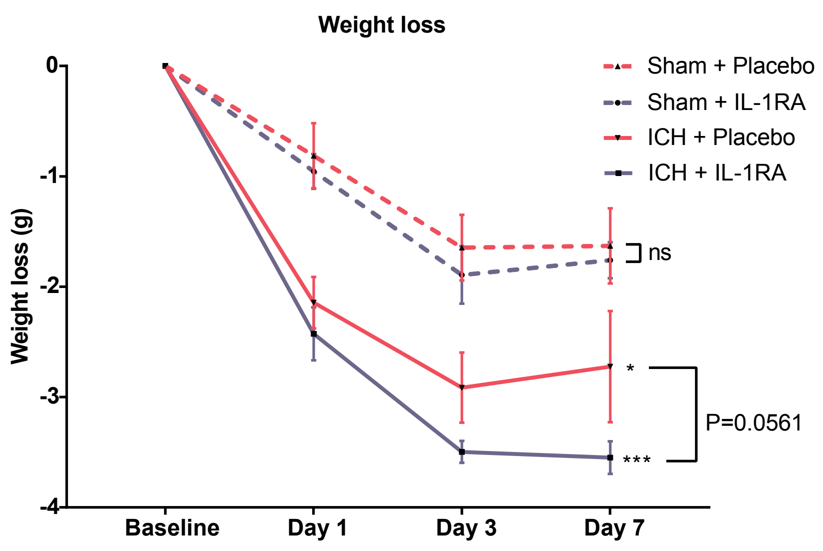

Fig. S5. Mice lose weight following intracerebral haemorrhage and there is a trend to increased weight loss in animals treated with Interleukin-1 receptor antagonist. Experimental design consisted of a placebo or an IL-1 receptor antagonist (IL-1RA) treatment regimen of 10 μg intrastriatal injection followed by a 100 mg kg^{-1} subcutaneous dose, prior to ICH induction, followed by another 100 mg kg^{-1} subcutaneous dose 6, 24, 30, 48 and 54 hours later. Weight was measured the same day of a rotarod behavioural assessment and presented as grams lost from pre-surgery weight. * = $P<0.05$ & *** = $P<0.0005$ vs sham-operated controls of same treatment group. ns = not significant, determined by mixed-effects model.

Disease Models & Mechanisms • Supplementary information
